# Supplementary material for: Molecular Cytological Analysis and Specific Marker Development in Wheat-Psathyrostachys huashanica Keng 3Ns Additional Line with Elongated Glume
Source: Int J Mol Sci. 2023 Apr 4;24(7):6726. doi: 10.3390/ijms24076726 (PMC10094845; doi:10.3390/ijms24076726)
Supplement: Supplementary file 1 [file ijms-24-06726-s001.zip › Supplemental Figures.pdf]

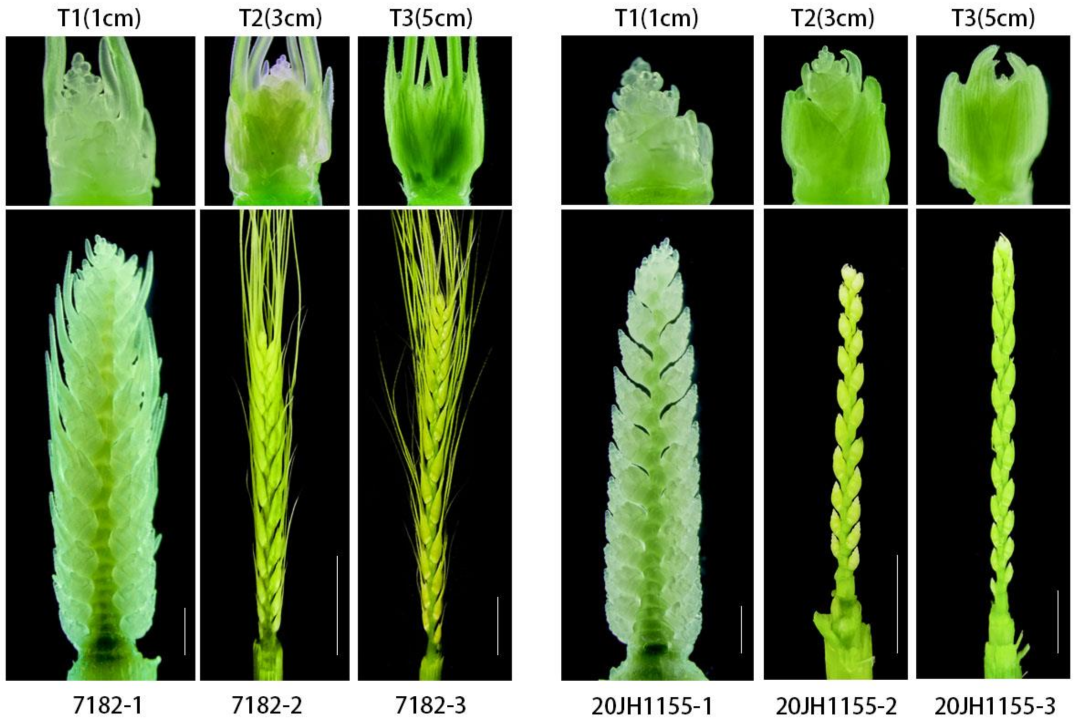

Supplemental Figure S1. Developmental stages of spikes used for sampling in this study. T1, T2, T3 correspond to 3 developmental points (Feekes 6,7,8) at jointing stage and booting stage.

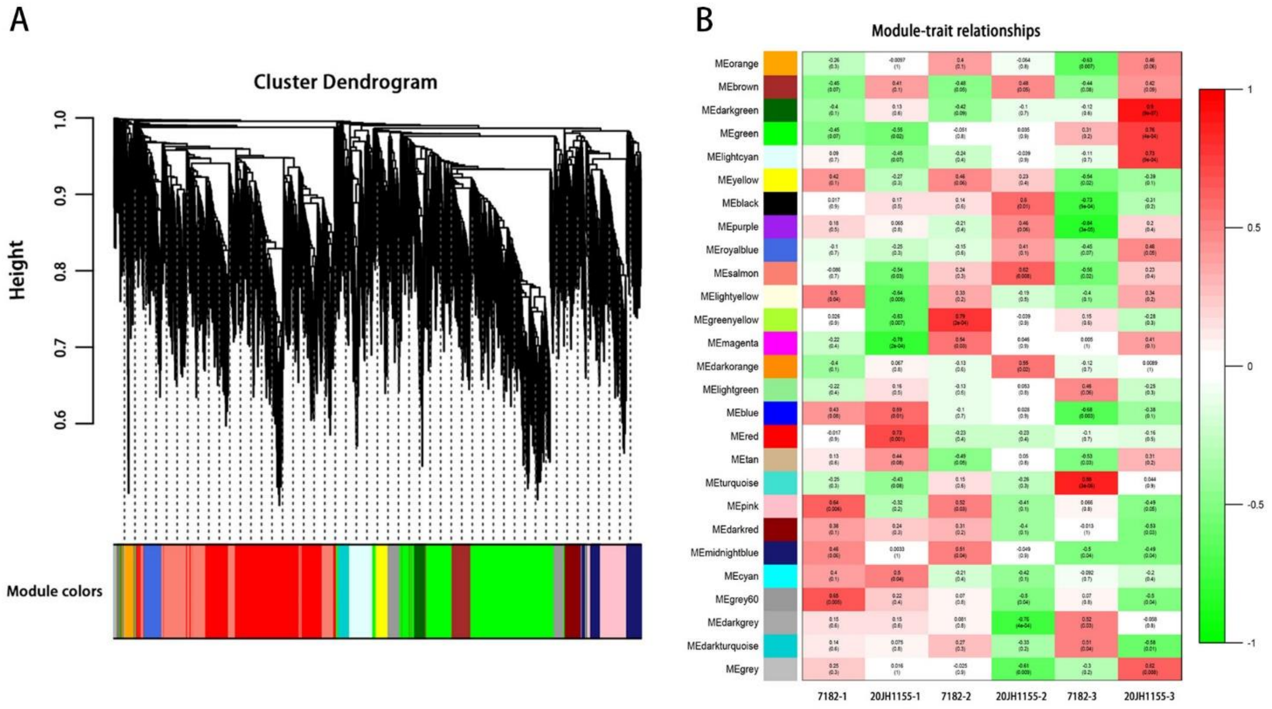

Supplemental Figure S2. Clustering and module identification of co expressed genes.

(A) Hierarchical clustering tree of DEGs by WGCNA. A total of 27 modules were divided according to gene function. **(B)** Module–time relationships. The value above the box represents the correlation coefficient between the module and time as well as the value below represents the significance between them.
